# Heterogeneity of cytotoxic T cell infiltration in breast and colorectal cancer

**DOI:** 10.1186/2051-1426-3-S2-P112

**Published:** 2015-11-04

**Authors:** James Ziai, Houston Gilbert, Oded Foreman, Jeffrey Eastham-Anderson, Felix Chu, Mahrukh Huseni, Jeong M Kim

**Affiliations:** 1 Genentech, Inc., South San Francisco, CA, USA

## Background

The prognostic value of intra-tumoral cytotoxic T cells has been demonstrated in multiple tumor types. However, immune infiltrate heterogeneity can lead to potentially significant misrepresentations of marker prevalence in routine histological sections. We examined step sections of breast and colorectal cancer samples for CD8+ T cell prevalence by standard chromogenic immunohistochemistry to determine marker variability and inform practice of T cell biomarker assessment in formalin-fixed, paraffin-embedded (FFPE) tissue samples.

## Methods

Serial sections were cut at approximately 25 um intervals from 13 primary colorectal and 12 breast carcinoma (8 ductal adenocarcinoma, 4 medullary) FFPE samples until blocks were exhausted and stained for CD8 using DAB-based chromogenic immunohistochemistry. Stained sections were digitally imaged and CD8+ lymphocytes within defined regions of interest (ROI) including the tumor and surrounding stroma were enumerated. Using a linear model/ANOVA framework, statistical analyses of CD8+ cell count variance between patients as well as between levels within a patient sample were performed. Means, medians, and dispersion values of percent CD8 positive cells (CD8 cells/total ROI cells) were calculated. Similarity of CD8 prevalence between slices of a given sample was measured by percent of variability accounted for by slice as a factor in a linear model and an intra-class correlation coefficient (ICC) ranging from zero (no similarity) to 1 (identical).

## Results

The mean and median CD8+ cell percentages varied between breast ductal adenocarcinoma (mean 11.32%, median 6.35) and colonic adenocarcinoma samples (mean 3.91%, median 2.88%) and were highest in medullary breast carcinoma (mean 25.32%, median 18.55%). However, minimal variation in cytotoxic T cell counts between levels within any given tumor block was observed. Percent variability (%) in CD8 counts between step sections from colonic adenocarcinoma (0.1%), medullary breast carcinoma (0.1%) and ductal adenocarcinoma (0.1%) blocks were minor. ICC calculated for percent CD8 positive cells between levels of a sample was 0.99 for both tumor types. Resampling-based methods show CD8 as reliable marker for classifying patients as CD8-positive or negative over a range of cut-offs.

## Conclusions

Our results show that CD8+ T cell distribution is highly homogeneous within a standard tissue sample in both colorectal and breast carcinomas. As such, cytotoxic CD8 T cell prevalence by immunohistochemistry on a single level can be considered representative of cytotoxic CD8 T cell infiltration for the entire tumor section within the block. These findings support the technical validity of biomarker strategies relying on CD8 immunohistochemistry in FFPE tumors.

